# Exploring the Complexities of TGF-β Signaling in Keloids: Beyond the Classical Smad Pathway

**DOI:** 10.3390/ijms27083600

**Published:** 2026-04-17

**Authors:** Jiao Mo, Hui Huang, Baochen Zhu, Ruiheng Liao, Wei Li, Yange Zhang

**Affiliations:** Department of Plastic and Burns Surgery, West China Hospital, Sichuan University, Chengdu 610041, China; mojiao202010@163.com (J.M.);

**Keywords:** keloid, TGF-β, non-Smad signaling, fibrosis, targeted therapy

## Abstract

Keloid is a benign skin disease with excessive growth of fibroblasts, characterized by too much abnormal extracellular matrix deposited in the dermis. It is generally believed that transforming growth factor-β (TGF-β) is the core cytokine that causes keloid. Previously, it was thought that its pathogenic effect was mainly attributed to the classical Smad-dependent pathway. It directly shuttles signals to the nucleus to trigger pro-fibrotic gene transcription. However, accumulating evidence now points to the equally vital role of Smad-independent signaling. Unlike the direct nuclear translocation of Smads, these alternative pathways transmit signals through rapid intracellular kinase cascades. They jointly direct the proliferation, migration, anti-apoptosis, fibrogenesis, and chronic inflammation of fibroblasts in keloids. This review attempts to comprehensively clarify the molecular processes regulated by TGF-β through non-Smad pathways (such as MAPK, PI3K/Akt, Rho GTPase, Wnt/β-catenin, JAK/STAT). Translating these non-Smad insights helps to overcome the high recurrence rates of traditional therapies. Targeting these specific molecular hubs through combination and precision therapies serves to reprogram the fibrotic microenvironment.

## 1. Introduction

Keloids represent a substantial therapeutic dilemma in dermatologic and reconstructive medicine. These lesions manifest as benign yet aggressively growing fibroproliferative dermal tumors. They exhibit malignant tumor-like behaviors, including invasion beyond original wound boundaries, a high recurrence rate post-excision, and rarely spontaneous regression. Keloids bring functional impairment, cosmetic disfigurement, and considerable psychological distress to the patients. Due to its notorious resistance to conventional therapies, such as surgery, corticosteroid injections, and radiation, keloids also pose a persistent clinical challenge [1]. Accordingly, an urgent, unmet clinical need for novel targeted treatments remains. The prevalence of Keloids varies globally, with a predominant effect on highly pigmented and younger populations [2]. Despite this variation, the universal pathological features include excessive fibroblast proliferation, dysregulated apoptosis, disorganized extracellular matrix (ECM) deposition, and a self-sustaining inflammatory microenvironment [3]. Research indicates that glycolytic reprogramming (via the NEDD4L/YY1/HK2 axis) [4], broad systemic immune activation [5], and mechanical stress transduced by sensors like Piezo1 [6,7], collectively force fibroblasts into a pro-fibrotic senescent state [8]. Local inflammatory mediators, such as mast cell-derived granzyme B, actively liberate matrix-stored transforming growth factor-beta (TGF-β) [9]. Among the myriad molecules involved, TGF-β has unequivocally emerged as the master regulator driving the fibrotic cascade in keloid pathogenesis.

TGF-β constitutes a multifunctional superfamily of cytokines. It’s able to elicit the phenotypic transformation of normal fibroblasts. This superfamily comprises over 30 structurally homologous cytokines, notably three principal isoforms (TGF-β1, β2, and β3). As a master regulatory network, it governs fundamental processes of cellular growth, differentiation, and ECM homeostasis [10]. The pivotal role of TGF-β in the pathogenesis of keloids was first established in the early 1990s. Seminal research demonstrated that the number of fibroblasts and the level of TGF-β in keloid tissue are significantly increased compared to normal skin [11]. This landmark finding established TGF-β, specifically the TGF-β1 isoform, as the principal driver of the fibroproliferative response. In keloids, aberrant TGF-β overexpression arises from a complex multicellular network. Activated fibroblasts sustain continuous TGF-β production via autocrine loops [12], while infiltrating M2 macrophages [13,14] and keratinocytes [15] upregulate its paracrine signaling. The ECM serves as a dynamic reservoir, where syndecan-1 regulates latent TGF-β activity [16]. Plasma-derived exosomal circular RNAs can further activate TGF-β pathways in normal fibroblasts [17]. 

Historically, the pathogenic role of TGF-β in fibrosis is predominantly attributed to its canonical Smad-dependent signaling pathway [10]. However, therapeutic targeting of the classic Smad pathway is extremely challenging. TGF-β is both a key regulator of fibrosis and an indispensable guardian of tissue homeostasis. Systemic inhibition of this pleiotropic pathway could lead to severe toxic effects on the body rather than specific tissues. Side effects include impaired wound healing, mucosal inflammation, and cardiac valvopathy resulting from the disruption of TGF-β signaling in endothelial and immune cells. In addition to these direct target effects, redundancy and interplay within the fibrotic signaling network also pose a significant obstacle [18]. Consequently, these therapeutic limitations have redirected research focus toward Smad-independent signaling networks. Alternative non-canonical pathways, including MAPK, PI3K/Akt, Rho GTPase, Wnt/β-catenin, JAK/STAT, are essential mediators of keloid pathogenesis and may serve as more specific pharmacological targets [19]. 

Unlike previous literature that often focuses on isolated linear cascades, this article introduces a network-centered view of TGF-β signaling in keloids. By systematically integrating mechanical transduction, epigenetic modulation, and canonical/non-canonical crosstalk, the review aims to comprehensively dissect the TGF-β/non-Smad pathways in keloids. Ultimately, establishing this holistic conceptual framework will help provide new translational strategies for developing next-generation, precision anti-fibrotic therapies.

## 2. Canonical TGF-β/Smad Signaling 

**Figure 1:**
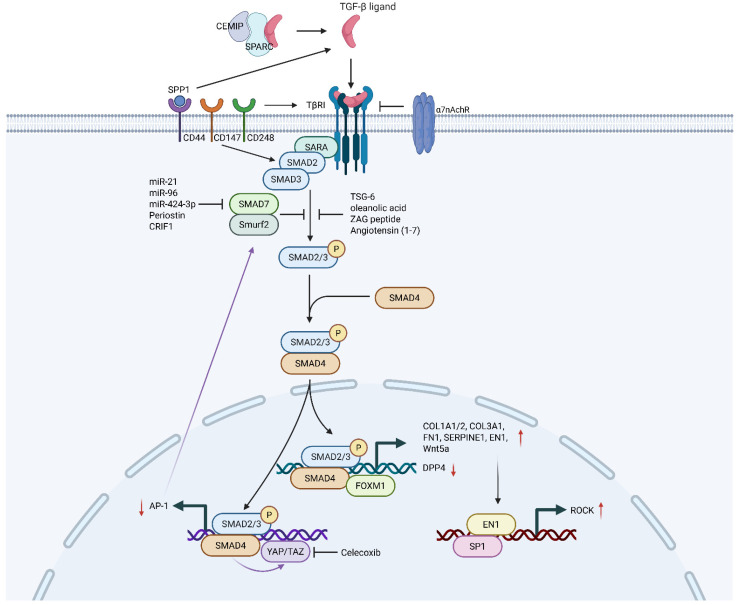
The classical TGF-β/Smad pathway. It kicks into action when TβR triggers the team-up between R-Smads and Smad4, forming a molecular tag-team that marches straight into the nucleus to crank up the expression of countless genes associated with fibrosis. Notably, miR-21, miR-96, miR-424-3p, periostin, and CRIF1 are able to suppress the inhibitory action of Smad7 on Smad. In contrast, TSG-6, oleanolic acid, ZAG peptide, and angiotensin (1–7) serve to inhibit the phosphorylation of Smad2/3, thereby preventing the advancement of keloid scarring. TGF-β/Smad3 induces the transcription factor EN1, which in turn upregulates ROCK-related genes via the EN1-SP1 axis. In addition, TGF-β/Smad3 activates YAP/TAZ in skeletal muscle fibrosis. YAP/TAZ in turn positively regulates TGF-β signaling through the AP-1/Smad7 axis. In the schematic diagram, red upward arrows (↑) represent upregulation, red downward arrows (↓) represent downregulation, standard arrows (→) indicate activation, and blunt-ended arrows (─|) indicate inhibition. Created with BioRender.com. Abbreviation: AP-1, activator protein 1; CD, cluster of differentiation; CEMIP, cell migration-inducing and hyaluronan-binding protein; EN1, engrailed-1; FOXM1, forkhead box protein M1; ROCK, Rho-associated protein kinase; SP1, specificity protein 1; SPARC, secreted protein acidic and rich in cysteine; SPP1, secreted phosphoprotein 1; YAP/TAZ, Yes-associated protein/transcriptional coactivator with PDZ-binding motif. Created with BioRender.com. https://BioRender.com/va1j40s (accessed on 29 March 2026).

The classical or canonical TGF-β signaling cascade is activated when a TGF-β ligand binds to a heteromeric complex of transmembrane serine/threonine kinase receptors, namely the type I receptor (TβRI) and type II receptor (TβRII). Figure 1 details the molecular architecture of the canonical TGF-β cascade. Ligand interaction induces TβRII phosphorylation and TβRI activation. Activated TβRI subsequently triggers C-terminal SSXS motif phosphorylation in Smad2 and Smad3. These phosphorylated R-Smads then form heteromeric complexes involving the common mediator Smad (Co-Smad), Smad4. This oligomeric assembly is imported into the nucleus to function as a transcription factor, binding to specific DNA sequences in the promoters of target genes, often in cooperation with other transcription factors and co-activators [20]. This nuclear translocation and transcriptional activation lead to the upregulation of pro-fibrotic genes, including those encoding type I and type III collagen, fibronectin, and plasminogen activator inhibitor-1 (PAI-1) [21]. Importantly, the pathway shapes a self-sustaining chronic inflammatory microenvironment. By way of example, TGF-β1/Smad signaling suppresses DPP4 expression, leading to CXCL12 accumulation that continuously recruits inflammatory cells via the CXCR4 receptor [22]. The pathway is tightly regulated by inhibitory Smad7. Smad7 serves as a universal repressor of signals by competing with Smad2/3 for receptor binding, targeting the receptors for degradation, and recruiting phosphatases. Notably, the expression or function of Smad7 is often compromised in keloids through converging upstream signals. Its downregulation is synergistically driven by overexpressed microRNAs (miR-21, miR-96, and miR-424-3p) [23,24,25], Periostin-integrin αV signaling [26], and CRIF1 [27]. Collectively, these diverse regulators dismantle the Smad7-mediated negative feedback loop, ensuring the sustained hyperactivation of TGF-β/Smad signaling (Figure 1).

The hyperactivity of the Smad pathway in keloids is underpinned by a multilayered regulatory network that amplifies signal transduction and receptor stability. At the transcriptional level, transcription factors FOXM1and TWIST1 are highly expressed in keloids, while FOXO4 is downregulated. Downregulated FOXO4 removes the inhibition on TRAF3IP2, positively activating the TGF-β1/Smad pathway [28]. FOXM1 is an effective amplifier of the TGF-β1 signal, and TWIST1 stabilizes MEF2A protein to upregulate TβRI gene transcription [29,30]. And the deubiquitinating enzyme USP11 markedly stabilizes TβRII protein levels through direct binding and deubiquitylation [31]. The transmembrane proteins CD44, CD147, and CD248 are all key positive regulatory factors of the TGF-β1/Smad2 pathway [32,33,34]. Elevated CEMIP promotes the pathway via binding to SPARC [35]. In addition to approaches targeting TGF-β ligands and receptors, recent studies have revealed that numerous drugs, including TSG-6 [36], oleanolic acid [37], Angiotensin (1–7) [38], and short peptides derived from zinc-α2-glycoprotein [39], exert an anti-keloid effect by inhibiting the phosphorylation of SMAD2/3 (Figure 1). Nevertheless, current precision therapies targeting the TGF-β/Smad pathway are mostly in the research stage. The first-line choice in clinical practice remains corticosteroid injections, in conjunction with other medications or physical therapy.

## 3. Non-Smad Signaling Networks

**Figure 2:**
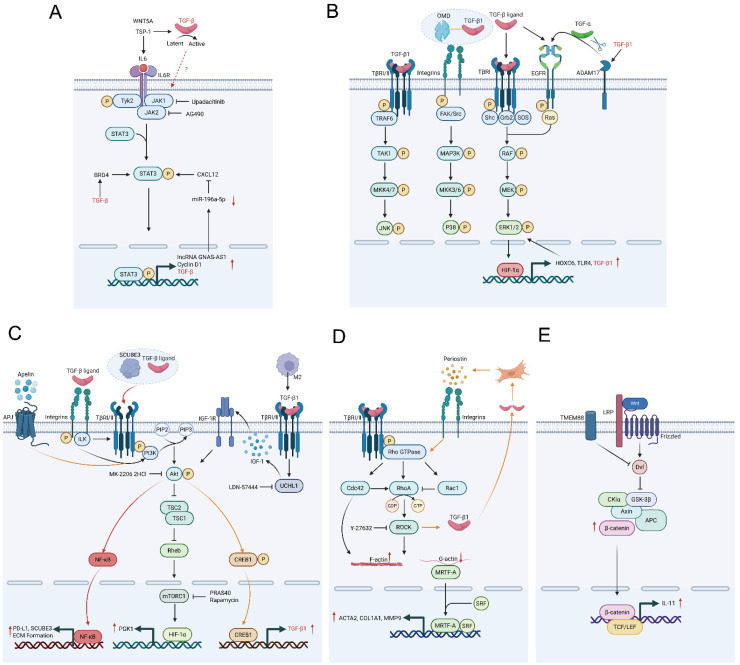
Schematic representation of non-SMAD-related signaling pathways implicated in keloid pathogenesis. (**A**) In keloids, IL6 is upregulated, binds to IL6R, activates JAK kinase, STAT3 is recruited and phosphorylated, transported into the nucleus, and binds to specific DNA sequences to activate a series of fibrotic target genes (lncRNA GNAS-AS1, Cyclin D1, TGF-β). lncRNA GNAS-AS1 downregulates miR-196a-5p through ceRNA, relieving its inhibition on CXCL12. The specific connection between JAK/STAT upstream and TGF-β remains unclear. (**B**) TGF-β ligands can activate the MAPK cascade (JNK, p38, ERK1/2) through TβR, integrin receptors, and EGFR. ERK1/2 enters the nucleus to promote the transcription factor HIF-1α to target genes, promoting the generation of HOXC6, which in turn upregulates ERK1/2, forming a positive feedback loop. (**C**) There are multiple upstream pathways for PI3K/Akt: Apelin binds to the APJ receptor, TGF-β binds to integrin receptors, the complex of SCUBE3 and TGF-β binds to TβR, and IGF-1 binds to IGF-1R. Activated Akt directly phosphorylates the inhibitor of TSC2, relieving its inhibition on the small G protein Rheb, thereby activating mTOR1. mTOR1 phosphorylates HIF-1α, promoting the expression of PGK1. Apelin/APJ activates CREB1 by Akt to promote the transcription of TGF-β1. Akt can also activate the NF-κB transcription factor, which is the core hub connecting inflammation and fibrosis. (**D**) TGF-β activates the Rho GTPase family (CDC42, Rac1, RhoA), with Cdc42 and RhoA working together and Rac1 antagonizing RhoA. RhoA activates the downstream kinase ROCK, driving the polymerization of G-actin to form F-actin stress fibers. The consumption of G-actin causes it to dissociate from MRTF-A, and MRTF-A is transported into the nucleus to bind with SRF, promoting the transcription of fibrotic genes such as ACTA2, COL1A1, and MMP9. Additionally, Periostin can stimulate the Rho/ROCK pathway through integrin receptors to stimulate TGF-β secretion, and TGF-β in turn promotes the production of Periostin. (**E**) The Wnt pathway stabilizes and accumulates β-catenin by disrupting the integrity of the degradation complex composed of Axin, APC, GSK-3β, and CK1. β-catenin enters the nucleus and, together with the co-transcription factor TCF/LEF, promotes fibrogenic gene expression. In this diagram, red upward (↑) and downward (↓) arrows denote upregulation and downregulation, respectively; standard arrows (→) indicate activation, and blunt-ended arrows (─|) indicate inhibition. Created with BioRender.com. Abbreviations: AKT, protein kinase B; APJ, apelin receptor; CREB1, cAMP-responsive element binding protein 1; ERK, extracellular signal-regulated kinase; ILK, integrin-linked kinase; JAK, Janus kinase; JNK, c-Jun N-terminal kinase; mTORC1, mechanistic target of rapamycin complex 1; NF-κB, nuclear factor kappa B; p38, p38 mitogen-activated protein kinase; PI3K, phosphoinositide 3-kinase; Rho, Ras homolog; ROCK, Rho-associated protein kinase; STAT3, signal transducer and activator of transcription 3; Wnt, Wingless/Integrated. Created with BioRender.com. https://BioRender.com/tswl5by (accessed on 26 March 2026).

The intricate network of TGF-β signaling extends far beyond the canonical Smad pathway, engaging multiple non-Smad cascades of keloids (as shown in Figure 2). They are woven together throughout the entire keloid development process, from the early inflammatory spark to the later fibrosis that keeps the disease self-sustaining. 

### 3.1. NF-κB and JAK/STAT Pathways in the Inflammatory Initiation Phase 

Keloids originate from an abnormal and persistent inflammatory response, which establishes a self-sustaining microenvironment conducive to fibrosis. In this inflammatory initiation phase, the NF-κB and JAK/STAT pathways act as foundational drivers, not only amplifying inflammatory signals but also directly engaging with the core fibrotic machinery. The NF-κB pathway serves as a master inflammatory switch. Its aberrant activation in keloids is often triggered by damage-associated molecular patterns (DAMPs) and hypoxia. For instance, Mortalin overexpression stimulates both the NF-κB and TGF-β via the IL-1α receptor [40]. HIF-1α can also concurrently initiate the TLR4/MyD88/NF-κB inflammatory cascade and TGF-β signaling under hypoxic stress [41]. Those co-activation patterns reveal NF-κB’s role in bridging initial injury signals to the TGF-β axis, thereby setting the stage for disease progression.

Concurrently, the JAK/STAT pathway, particularly STAT3, functions as a signal amplifier and integrator, a complex topological network explicitly detailed in Figure 2A. In fibrotic diseases, STAT3 is overactivated, and this activation is TGF-β-dependent, co-mediated by multiple kinases such as JAK, SRC, c-ABL, and JNK. TGF-β may activate STAT3 through epigenetic factor BRD4. BRD4 drives STAT3 phosphorylation, which in turn regulates downstream targets such as c-Myc and KLF2/KLF4 [42]. The absence or inhibition of STAT3 itself significantly weakens the fibroblast’s response to TGF-β and effectively alleviates fibrosis in animal models [43]. Pharmacologically, JAK1/JAK2 inhibitors (Upadacitinib, AG490) can downregulate TGF-β1 expression and markedly restrain keloid fibroblasts (KFs) [44,45]. Importantly, STAT3 activation is not exclusively downstream of TGF-β. It can be independently ignited by parallel pathways such as the Periostin/JAK/STAT axis [46], the TSP1/IL-6/JAK2/STAT3 cascade [47], or the paracrine WNT5A/IL-6 axis from keratinocytes [48]. Omics data further support that the STAT3 pathway constitutes a core pathogenic module potentially governed by independent epigenetic programming [49]. Once activated, STAT3 can perpetuate its own signaling through STAT3/GNAS-AS1/miR-196a-5p/CXCL12/STAT3 feedback loops [50], cementing a chronic inflammatory state.

In brief, during the inflammatory initiation phase, NF-κB and JAK/STAT pathways operate in concert and through multiple crosstalk mechanisms with TGF-β signaling. While their functional synergy in promoting keloid fibrosis is evident, the precise molecular dialogue initiating this collaboration warrants further investigation. This foundational crosstalk effectively converts acute injury signals into a chronic pro-fibrotic microenvironment, paving the way for subsequent fibroblast activation.

### 3.2. Signaling Networks in the Activation and Proliferation Phase

Following the establishment of a chronic inflammatory microenvironment, resident fibroblasts enter a state of hyper-activation and uncontrolled proliferation. This phase is predominantly driven by the MAPK and PI3K/Akt/mTOR pathways. They function not merely as parallel effectors but as integrated components of a TGF-β-centric signaling network, engaging in extensive bidirectional crosstalk.

#### 3.2.1. The MAPK Cascade

The MAPK family is an amplifier and modulator of TGF-β signaling, an amplification network explicitly mapped in Figure 2B. The MAPK family represents a group of serine/threonine protein kinases that control crucial cellular functions like cell growth, specialization, stress management, and programmed cell death [51]. TGF-β activates ERK1/2 via TβRI-mediated Shc-Grb2-SOS-Ras cascade or through EGFR transactivation. MEK/ERK pathway blockade reduces collagen production and triggers programmed cell death in KFs [52,53]. Similarly, stress-responsive JNK and p38 branches are activated by TGF-β via upstream kinases like TAK1 [54]. Besides directly activating the Ras/ERK pathway via intracellular adaptor proteins, TGF-β can also initiate ERK signaling through receptor transactivation. Le and Fan discovered that ADAM17 cleaves and activates the membrane-bound precursor of the EGFR ligand TGF-α, thus triggering the EGFR/ERK signaling cascade. TGF-β1 upregulates ADAM17 expression and establishes a self-reinforcing cycle of TGF-β/ADAM17/EGFR/ERK [55]. Critically, factors like osteomodulin (OMD) can synergize with TGF-β1 to ensure sustained p38 activation [56], and BMP1 concurrently stimulates both p38 and ERK cascades in KFs [57]. Those demonstrate how non-Smad pathways amplify TGF-β-driven signaling. Furthermore, TGF-β1 can utilize the ERK pathway to trigger protective autophagy, a parallel survival signal that complements its Smad-mediated effects. In summary, these diverse receptor-mediated events converge to rapidly and persistently activate the MAPK axis. Such sustained activation represents its indispensable role in the TGF-β-driven fibrotic program.

#### 3.2.2. The PI3K/Akt/mTOR Axis

The PI3K/Akt/mTOR axis forms an essential co-regulatory circuit with TGF-β signaling. It governs cellular proliferation, viability, and metabolic processes [58]. Figure 2C shows how multiple upstream signals converge on the Protein Kinase B (Akt) hub to drive downstream effectors and self-amplifying feedback loops. TGF-β can activate PI3K directly or indirectly via integrin engagement. Subsequent phosphorylation of Akt activates mTORC1, leading to increased protein synthesis, proliferation, and autophagy dysregulation. Ultimately, KFs are activated [59]. Beyond classic rapamycin, PRAS40 can directly suppress the activation of mTORC1 in hypertrophic scar (HS) [60]. This indicates that mTOR is an important target in skin fibrosis. TGF-β/Smad pathway can also upregulate the transcription factor Runx2, which in turn positively regulates the PI3K/AKT pathway, creating a feed-forward signal [61]. Likewise, the hypoxic core protein SCUBE3 forms a functional complex with TGF-β ligand to selectively activate the PI3K/AKT/NF-κB cascade [62]. Those illustrated how non-Smad pathways can amplify TGF-β signal output. Pharmacologically, combined inhibition of both PI3K/Akt/mTOR and TGF-β/Smad pathways yields synergistic anti-fibrotic effects, underscoring their functional interdependence [63,64]. In addition, the PI3K/Akt/mTOR axis is a key node where the microenvironment modulates cellular sensitivity to TGF-β. M2 macrophages in the keloid microenvironment induce fibroblasts to highly express the deubiquitinating enzyme UCHL1 by secreting TGF-β1. UCHL1, by promoting IGF-1 signaling, aberrantly activates the Akt/mTOR/HIF-1α pathway to drive fibrosis [65]. Most significantly, the PI3K/Akt pathway can act upstream to initiate the TGF-β signal itself. The hypoxia-induced Apelin/APJ axis activates PI3K/Akt, resulting in CREB1 phosphorylation. Phospho-CREB1 directly binds the TGF-β1 gene promoter and transactivates it. This complex signaling pathway establishes a powerful self-reinforcing loop [66]. 

To summarize, during the proliferation phase, the MAPK and PI3K/Akt/mTOR pathways are deeply embedded within the TGF-β signaling network. They are activated by TGF-β, which in turn regulates TGF-β’s expression, stability, and contextual specificity. This dense web of crosstalk ensures a robust, self-reinforcing activation state of KFs, making these non-Smad pathways critical targets for disrupting the fibrotic cascade at its root.

### 3.3. Signaling Networks in the Matrix Remodeling and Maintenance Phase

Following the hyper-activation and proliferation of fibroblasts, keloid pathogenesis enters a decisive matrix remodeling and persistence phase. This stage is characterized by the translation of biochemical signals into irreversible structural changes, where pathways responsive to mechanical force and cellular memory become dominant. Their core function is to perpetuate the fibrotic phenotype, ensuring disease chronicity through sustained ECM deposition, contraction, and transcriptional reprogramming. 

#### 3.3.1. The RhoA/ROCK Axis

The Rho GTPase family, particularly RhoA/ROCK, constitutes the pivotal intracellular hub that translates mechanical environment and TGF-β signaling into sustained cytoskeletal action [67]. RhoA, Rac1, and Cdc42 belong to the small GTPases of the Rho family. This family primarily modulates the dynamic rearrangement of the cytoskeleton [68]. RhoA/ROCK signaling plays a crucial role in TGF-β-induced stress fiber formation, acquisition of a myofibroblast phenotype, and cellular contractility. In TGF-β-triggered cell migration, the Rho/ROCK pathway specifically mediates the mechanical invasiveness essential for amoeboid movement by inducing cytoskeletal contraction [69]. The RhoA/ROCK-MRTF-A axis is the primary pathway responsible for mediating the fibrotic phenotypes induced by TGF-β1 [70]. In mesenchymal stem cells, RhoA and Cdc42 act synergistically to mediate TGF-β-induced α-SMA expression, while Rac1 plays an inhibitory role. The central mechanism involves RhoA/Cdc42 driving cell contraction to increase F-actin levels, which facilitates MRTF-A movement into the nucleus, causing α-SMA transcription [71]. Figure 2D visually details this mechanical conversion network. Beyond execution, this pathway actively amplifies the TGF-β signal. The TGF-β/Smad3-induced transcription factor EN1 upregulates cytoskeletal and ROCK-related genes via the EN1-SP1 axis, priming cells for enhanced mechanical responsiveness [72] (Figure 1). Furthermore, a potent positive feedback loop exists. Periostin, induced by inflammatory factors IL-4/IL-13, activates RhoA/ROCK, which stimulates further TGF-β1 secretion, which in turn enhances Periostin synthesis [73]. Thus, Rho GTPases transform a transient TGF-β signal into a self-sustaining cycle of contraction and matrix production, cementing the fibrotic state.

#### 3.3.2. The Hippo/YAP/TAZ Pathway

The Hippo pathway effectors YAP/TAZ are central integrators of mechanical and biochemical cues, and their interaction with TGF-β in keloids is complex and stage-specific [74]. In skeletal muscle fibrosis, TGF-β1 activates YAP/TAZ via its canonical SMAD3 pathway [75]. Once activated, YAP/TAZ establishes a positive feedback loop by repressing the AP-1/Smad7 inhibitory axis, thereby sustaining and amplifying the initial TGF-β1 signal [76]. Research found that celecoxib effectively inhibits TGF-β1 mediated myofibroblast differentiation by specifically suppressing YAP/TAZ protein expression and nuclear migration [77] (Figure 1). Intriguingly, in established keloids, a study found that high nuclear expression of YAP becomes unresponsive to further increases in matrix stiffness or TGF-β stimulation. While their TGF-β/Smad signaling remains functionally intact, it is not hyperactivated [78]. This suggests that in the persistence phase, YAP/TAZ may transition from a dynamic regulator to a constitutively active permissive factor. It can maintain a cellular state conducive to fibrosis without continuous TGF-β stimulation, which contributes to therapeutic resistance.

#### 3.3.3. The Wnt/β-Catenin Pathway

The Wnt/β-catenin pathway is a key driver of fibrotic memory, engaging in extensive, bidirectional crosstalk with TGF-β to lock in pathological gene expression programs [79]. A primary dialogue mechanism is ligand-mediated induction. TGF-β stimulates the transcription of Wnt ligands within KFs (Figure 1), which then activate the Wnt/β-catenin pathway in an autocrine/paracrine manner [80]. The activation of this pathway results in the stabilization of β-catenin, a key downstream event mediated by the suppression of GSK-3β. Nuclear β-catenin then partners with TCF/LEF to promote the expression of fibrogenic and proliferative genes [81]. Figure 2E explicitly maps its nuclear translocation and integration into fibrotic transcriptional complexes. This interaction is functionally synergistic and amplified, as demonstrated in other fibrotic models where Wnt/β-catenin enhances TGF-β effects via TAK1 activation and IL-11 induction [82]. Therapeutically, whether with Pirfenidone, decoy receptors like sLRP6E1E2, or TMEM88 overexpression, one effectively dampens β-catenin activity while simultaneously quelling the TGF-β/Smad pathway. This emphasizes the intricate connection between the two pathways [83,84,85]. Crucially, Wnt/β-catenin also operates as a parallel, independent pro-fibrotic driver. Selective β-catenin engagement in follicular dermal sheath progenitor cells is sufficient to autonomously induce skin fibrosis and upregulate ligands such as BMP, FGF, and Notch [86].

Collectively, the matrix remodeling phase is defined by pathways that ensure the transition from active response to permanent change. Rho GTPases lock in the cellular contractile apparatus, Hippo/YAP-TAZ may lock in a permissive cellular state, and Wnt/β-catenin locks in the pro-fibrotic transcriptional program. All three are deeply engaged in reciprocal dialogue with the TGF-β signal, making the keloid phenotype self-sustaining and extraordinarily difficult to reverse. This explains the pronounced therapeutic resistance observed in late-stage keloids and emphasizes the necessity of targeting these phenotype-persistence mechanisms.

### 3.4. The Metabolic and Epigenetic Regulatory Layer

The inflammatory, proliferative, and matrix-remodeling phases described above are not transient events but are consolidated into a persistent pathological state. This sustained activation is underpinned by a deeper metabolic and epigenetic regulatory layer. The pathways within this layer do not merely respond to TGF-β. They fundamentally reshape the cellular context to perpetuate TGF-β signaling and lock in the fibrotic phenotype, explaining the disease’s resistance to resolution.

Integrin-Linked Kinase (ILK): In the stiffened keloid microenvironment, ILK acts as an integrator of mechanical (integrin) and chemical (TGF-β) signals, ensuring the sustained activity of the TGF-β network. Beyond its role in transducing matrix stiffness, ILK directly stabilizes the TGF-β receptor complex. ILK directly binds to TβRII and prevents its entry into the membrane raft-mediated ubiquitin-proteasome degradation pathway. Through this approach, the cellular sensitivity to TGF-β ligands is enhanced and prolonged [87]. This stabilization is functionally essential, as TGF-β1-induced myofibroblast differentiation is dependent on the activation of the ILK-PI3K/Akt signaling axis. The mechanism is further detailed in Figure 2B [88]. Thus, ILK may create a positive feedback loop. It is activated with matrix stiffness, stabilizing TGF-β receptors, which leads to stronger TGF-β signaling and further matrix production, cementing the fibrotic state.

Peroxisome proliferator-activated receptors (PPARs): PPARs, particularly PPARγ, function as endogenous brakes on the fibrotic program, and their downregulation in keloids releases this inhibition. Their agonists exert potent antifibrotic effects by multipronged interference with TGF-β signaling networks. Both pan-PPAR agonist IVA337 and the lysate of *Streptococcus thermophilus* can simultaneously regulate the canonical and non-canonical TGF-β signal [89]. The *S. thermophilus* lysate simultaneously curbs TGF-β1 mRNA level and β-catenin protein output by upregulating PPARγ [90]. More specifically, PPARγ agonist troglitazone inhibits the receptor tyrosine kinase Axl by upregulating miR-92b, resulting in the descending expression of TGF-β1 and its downstream fibrotic genes. It was confirmed that the PPAR-γ/miR-92b/Axl/TGF-β1 loop co-drives the fibrosis of keloids [91]. Those highlight PPARs as master regulators capable of damping multiple fibrotic pathways at once.

Notch Signaling: TGF-β and Notch signaling pathways exhibit intricate crosstalk during fibrosis [92]. A study by Huo et al. indicates interplay between TGF-β/Smad3 and Notch signaling cascades, which collectively facilitate OPA1-mediated mitochondrial fusion [93]. Interventions such as adipose-derived stem cell exosomes (ASC-Exos) concurrently diminish the protein expression of TGF-β2 and Notch-1 in KFs [94]. Notch signal inhibition was concomitant with a downregulation in TGF-β1 expression [95]. All those suggest that Notch and TGF-β are co-dependent components of a pro-fibrotic gene regulatory program. Notch is a significant, though underexplored, node in the keloid signaling network.

Transglutaminase 2 (TGM2): TGM2 operates as a key enzymatic effector that translates sustained TGF-β signaling into irreversible ECM stabilization. As a cross-linking enzyme, TGM2 is directly upregulated by TGF-β1 treatment alongside other ECM modifiers like lysyl oxidase-like protein 1, 2, 4, and lysyl hydroxylase 2 [96]. TGM2 expression significantly correlates with the expression of HIF-1A, IL-6, and FN1 in keloids [97], positioning it at the intersection of multiple pathological stimuli. Importantly, the functional crosstalk between TGM2 and TGF-β is bidirectional and conserved across fibrotic diseases. In systemic sclerosis, TGM2 inhibition reduces fibrotic markers comparably to TGF-β neutralization. And, exogenous TGF-β partially rescues the migration and contraction defects in TGM2-deficient fibroblasts. [98]. This establishes TGM2 not merely as a passive downstream target, but as an active regulator and amplifier within the TGF-β signaling network. 

Taken together, the ILK, PPAR, Notch, and TGM2 pathways constitute a regulatory stratum that sustains the fibrotic programme. They achieve this by stabilizing TGF-β reception (ILK), dysregulating endogenous inhibitory circuits (PPARs), locking in profibrotic cell fate (Notch), and executing irreversible matrix hardening (TGM2). These mechanisms lock the initial cellular activation into a metabolically favored, epigenetically ingrained, and structurally permanent phenotype. Consequently, this multi-layered stabilization drives the chronicity and therapeutic recalcitrance of keloids.

## 4. Crosstalk Between Smad and Non-Smad Signaling Networks

Recent studies have elucidated that in keloids, the canonical and non-canonical TGF-β pathways converge into a tightly knit, hierarchical network via key molecular nodal points. It accounts for the disease’s chronicity and pleiotropic manifestations. The representative molecular interactions driving the Smad and non-Smad crosstalk are demonstrated in Table 1. 

### 4.1. Direct Bidirectional Regulation and Mutual Amplification

One of the most prominent forms of crosstalk involves the direct modulation of Smad signaling by non-Smad pathways. For instance, MAPK pathways, particularly ERK, JNK, and p38, can phosphorylate Smad proteins at sites distinct from the TβRI-mediated C-terminal phosphorylation, thereby influencing Smad activity, stability, or nuclear translocation [99]. Inhibition of ERK1/2 was sufficient to block TGF-β1 induced Smad signaling and downstream fibrotic responses [100], and MKP-5 inhibition blocks TGF-β signaling via a JNK-dependent pathway [101]. The transcriptional effector of the RAS/MAPK pathway, RREB1 is indispensable for the TGF-β-activated SMAD protein to promote the expression of genes involved in EMT and fibrosis [102]. It exemplifies advanced integration by pre-binding to target enhancers (such as IL11, PDGFB, and SNAI1), with the TGF-β signal recruiting the SMAD4/INO80 complex to these pre-marked sites for activation [103]. On the other hand, the canonical pathway can also act as an initiator for reinforcing signals. For example, the TGF-β1/Smad signaling pathway directly upregulates the transcription factor EGR1. EGR1 increases the expression of NADPH oxidase 4 (NOX4), leading to a reactive oxygen species (ROS) burst that further drives the fibrotic progression of keloids [104]. Furthermore, LTBP4 and its companion protein Fibulin-4 can directly bind to cell surface Syndecan-2/3 receptors, initiating a pro-contractile and pro-synthetic signaling program mediated by FAK, ERK, and RhoA [105]. Thus, the direct bidirectional crosstalk between Smad and non-Smad modules fundamentally limits the efficacy of single-pathway inhibitors.

**Table 1:** Key crosstalk mechanisms between TGF-β/Smad and non-Smad pathways in keloids.

Key Node	Non-Smad Pathway	Crosstalk Mechanism with TGF-β/Smad	Refs
SDPR	ERK1/2 (MAPK)	ERK1/2-mediated Smad suppression; reduced fibroblast proliferation and ECM production	[100]
MKP-5	JNK (MAPK)	Essential signaling bridge; JNK-dependent TGF-β routing; requisite for fibrotic activation	[101]
RAS/RREB1	RAS cascade	Synergistic gene regulation with Smads; unmasking of primed enhancers; EMT promotion	[102]
Integrin/FAK	PI3K/AKT & ERK	Parallel activation with Smads; mechanotransduction and miRNA modulation; accelerated collagen deposition	[106,107]
WNT5A	Noncanonical WNT	Upstream extracellular trigger; latent TGF-β activation; dual initiation of Smad and non-Smad cascades	[108]
CPEB1/CPEB4	TAK1	Dual pathway regulator; simultaneous modulation of TAK1 and Smad activities	[109]
EGR1	NOX4 (Oxidative Stress)	Linkage of TGF-β to oxidative stress; NOX4-driven ROS production; positive feedback loop for fibrosis	[104]

Abbreviations: CPEB, Cytoplasmic polyadenylation element-binding protein; EGR1, Early growth response 1; FAK, Focal adhesion kinase; MKP-5, Mitogen-activated protein kinase phosphatase-5; NOX4, NADPH oxidase 4; RREB1, Ras-responsive element-binding protein 1; SDPR, Serum deprivation protein response; TAK1, Transforming growth factor-beta-activated kinase 1.

### 4.2. Key Molecules as Signal Integrators

The complexity of the fibrotic signaling network lies in key molecules that function as physical and functional signal integrators, receiving diverse inputs and coordinating multifaceted outputs. These hubs form a stratified hierarchy, cascading from the microenvironment down to post-transcriptional regulation. At the uppermost environmental tier, stressors and upstream orchestrators, such as hypoxic signals (HIF-1α/HOXC6), DAMPs (HMGB1), and guidance molecules (Slit1), ignite the network. They not only induce TGF-β expression but also synergistically activate parallel Smad and non-Smad (ERK, Akt, TAK1/MAPK) pathways [41,110,111,112]. These biochemical cues are physically coupled to the ECM through mechanotransduction hubs. For instance, WNT5A mechanically activates latent TGF-β in the ECM via cytoskeletal rearrangement [108]. This mechanical tension is then consolidated at the plasma membrane by focal adhesion scaffold proteins like Zyxin and Kindlin-2. Serving as dual-function integrators, Zyxin concurrently regulates FAK/PI3K/Akt and TGF-β pathways [107]. Kindlin-2 sustains cell survival by positively driving TGF-β/Smad signaling while simultaneously inhibiting Fas-mediated apoptosis [113]. Once transmitted intracellularly, this intricate dialogue is further amplified. The high expression of PDE4 in keloids synergistically enhances Smad3 and ERK1/2 signaling by impairing the Smad3 phosphatase PPM1A [114]. This intricate signaling dialogue is even fine-tuned at the post-transcriptional level. RNA-binding proteins CPEB1 and CPEB4 coordinately modulate the translation of key mRNAs within both Smad and TAK1/MAPK pathways [109]. Specifically, the downregulation of miR-3606-3p results in the coordinated upregulation of its three target genes, ITGAV, GAB1, and TGFBR2. Signaling initiated by ITGAV via the integrin/FAK axis is proven to unidirectionally activate the downstream AKT/ERK and SMAD2/3 pathways. GAB1 serves as a crucial adaptor for the PI3K/Akt and MAPK/ERK pathways, while TGFBR2 initiates the canonical Smad cascade. Upon receiving upstream integrin signals, these two parallel hubs collectively drive a potent cross-amplification loop [106]. These key molecules function as crucial hubs within the complex signaling network. Consequently, targeting these key node molecules, rather than inhibiting upstream cascades, promises more precise and efficacious therapies.

## 5. Functional Heterogeneities of TGF-β Subtypes

Traditionally, TGF-β signaling was viewed as a monolithic cascade. However, recent research pointed out a need to deconstruct its complex functions by examining ligand specificity and cellular heterogeneity. As a multifunctional cytokine family, mammalian TGF-β comprises three isoforms: TGF-β1, TGF-β2, and TGF-β3 [115]. In physiological scar formation, the P311 protein has been shown to synchronously upregulate the translation of all three TGF-β subtypes [116]. Studies on fibroblasts from keloids and HS have shown that TGF-β1 acts as a strong pro-fibrotic agent, while TGF-β3 demonstrates an opposing, suppressive effect [117]. Although typical keloid is predominantly driven by the TGF-β1/Smad signaling axis, whereas acne scars present with significant upregulation of TGF-β3 and no change in TGF-β1, with their fibrosis potentially being governed by the TGF-β3/Smad axis [118]. In keloid tissue, the principal drivers of fibrosis are the markedly upregulated TGF-β1, at approximately 10-fold, and TGF-β3, at approximately 2-fold, whereas the expression of TGF-β2 remains largely unchanged. TGF-β1 acts upon the RTK signaling pathway via SPRY1, TGF-β2 activates Wnt signaling, and TGF-β3 initiates HIF-1 signaling via EDN1[119]. This isoform-signaling specificity ensures that each subtype engages a unique transcriptional and cellular response network, which is actively translated into novel therapeutic concepts.

The well-established anti-fibrotic role of TGF-β3 has inspired innovative approaches. The delivery of modified TGF-β3 and IL-10 mRNA via adipose-derived stem cells (ADSCs) or the local, sustained release of engineered TGF-β3 protein during wound healing, both of which have shown promise in improving collagen architecture and reducing scarring in preclinical models [120,121]. And engineered delivery of miR-29a using ASC-Exos mitigates scar formation by directly targeting TGF-β2 to inhibit the Smad3 signaling pathway [122]. Furthermore, TGF-β signal output exhibits cellular heterogeneity, which is a fundamental source of signaling complexity in keloids. Research has found that POSTN+ fibroblasts enriched in scars are a high-activity TGF-β signaling center, whereas IGFBP2+ fibroblasts in normal skin are insensitive to it [123]. This suggests that the key to fibrosis may lie in the high sensitivity of specific cell subpopulations to TGF-β signals. Validating this concept, recent single-cell studies characterized the dominant fibrotic subpopulation in keloids. Within these specific cells, the TGF-β and Eph-ephrin pathways operate in synergy. This cooperative network is orchestrated by the transcriptional hub composed of TWIST1-FOXO3-SMAD3 [124]. The functional heterogeneity of TGF-β, dictated by specific signaling programs, epigenetic regulators, and recipient cell identity, constitutes the complexity in pathological scarring.

## 6. Epigenetic Regulation of TGF-β Signaling

The pathogenesis of keloids transcends mere transient signaling activation and is deeply rooted in durable epigenetic reprogramming. This layer of regulation encompasses DNA methylation, histone modifications, and a vast network of non-coding RNAs (ncRNAs). It functions to inscribe the fibrotic program into the cellular repertoire, ensuring its persistence long after the initial wound stimulus has been resolved. For instance, the methylation-binding protein MeCP2 inhibits Smad7 expression through mediating methylation of the Smad7 promoter [125]. Lactic acid induces H3K18 lactylation in KFs. This modification directly upregulates the transcription of LTBP3, promoting TGF-β1 secretion [126].

**Figure 3:**
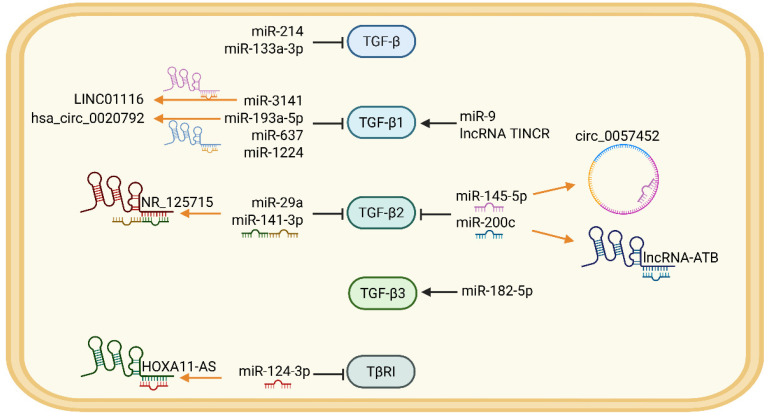
The TGF-β ligand and its receptor are regulated by related non-coding RNAs. The blank arrows indicate promotion, while the blank flat lines represent inhibition. lncRNAs and circRNAs downregulate miRNA through sponge adsorption, as shown in the orange arrows. Created with BioRender.com. https://BioRender.com/gn8a6ni (accessed on 29 March 2026).

The most extensive and intricate dimension of this regulation is orchestrated by the diverse families of ncRNAs. To visually unpack this multi-tiered control, Figure 3 delineates how various ncRNA networks specifically converge to regulate the TGF-β ligand and its receptor complex. The expression of TGF-β2, for example, is modulated by a complex competing endogenous RNA (ceRNA) network involving circ_0057452 and miR-145-5p [127]. This regulatory sophistication is exemplified by lncRNA-ATB, which promotes a pathogenic TGF-β2 autocrine loop by sequestering miR-200c and upregulating the transcription factor ZNF217 [128]. This regulatory sophistication extends to the anti-fibrotic TGF-β3, whose expression can be upregulated by miR-182-5p in inflammatory contexts [129]. Beyond ligand regulation, key ncRNAs function as nodal integrators that concurrently amplify multiple downstream pathways. miR-21, highly expressed in wound healing, simultaneously inhibits multiple targets such as Smad7, PTEN, and SPRY1/2, forming a regulatory network that synergistically enhances TGF-β/Smad, PI3K/Akt, and MAPK/ERK signaling [130,131]. More importantly, Nuclear miR-9 acts as a crucial cofactor to directly amplify TGF-β1-driven transcription. It promotes G-quadruplex formation and enhancer-promoter interactions, restructuring the 3D chromatin architecture [132]. The roles of these regulators are often complex and context-dependent. miR-141-3p has been identified to enhance the activity of the PI3K/Akt/mTOR pathway and target TGF-β2 mRNA [133,134]. Additional microRNAs continuously adjust this cascade through distinct mechanisms. While miR-1224-5p directly intercepts Smad3 mRNA [135], others act indirectly, such as miR-214 targeting the adenosine A2A receptor [136] and miR-133a-3p suppressing IRF5 [137]. Specifically, the availability of these microRNAs is dictated by a higher-order ceRNA network of long non-coding RNAs (lncRNAs) and circRNAs.

Revealing the breadth of this network, recent high-throughput sequencing analysis of HS revealed a complex regulatory network of lncRNAs and circRNAs. Notably, differentially expressed molecules such as AC048380.1 and LINC00299 exhibit a strong correlation with the expression of key TGF-β pathway genes, namely TGF-β3, SMAD7, and INHBA [138]. Many of these molecules are embedded within pathological positive feedback loops. TGF-β1 induces the expression of LINC00525. Activated LINC00525 adsorbs miR-29a-5p and promotes the synthesis of TGF-β1 [139]. lncRNAs typically function through the ceRNA mechanism. For example, lncRNA NR_125715, HOXA11-AS, and LINC01116, competitively bind to miR-141-3p and miR-29, miR-124-3p, and miR-3141, respectively, resulting in the elevated TGF-β1/2 and TβRI [140,141,142]. In addition to the ceRNA mechanism, lncRNA TINCR can also drive TGF-β1 expression by directly interacting with protein SND1 [143]. This network also incorporates endogenous braking mechanisms. LncRNA GAS5 can directly bind to Smad3 and promote its dephosphorylation by PPM1A, acting as an endogenous brake to inhibit TGF-β/Smad3 signaling and fibrosis [144]. And overexpression of COL1A2-AS1 induces fibroblast apoptosis by synergistically inhibiting the phosphorylation and activation of the TGF-β/Smad3 pathway and promoting the β-catenin expression [145]. Beyond linear transcripts, circRNAs further elevate this regulatory complexity. The hsa_circ_0000437/let-7f-5p/THBS1 axis constitutes a ceRNA network that finely regulates TGF-β2 activity and the progression of skin fibrosis [146]. Translatable circRNA like CircGLIS3(2), induced by TGF-β, represents the frontier of ncRNAs evolution. It simultaneously possesses dual functions of non-coding regulation and protein coding, greatly expanding the epitranscriptomic output dimensions of the TGF-β signal [147]. 

In addition to ncRNA networks, the epitranscriptomic landscape of TGF-β signaling is dysregulated in keloids, particularly at the level of N6-methyladenosine (m6A) modification [148]. m6A methyltransferase KIAA1429 is diminished in keloids, inducing fibrosis through stabilization of TGF-β1 mRNA [149]. Besides, METTL3 is highly expressed in HS and promotes fibrogenesis by way of m6A methylated GRAMD1B transcript [150]. The m6A reader protein HNRNPC enhances its own expression by stabilizing WDR77 mRNA, which in turn upregulates TGF-β and SMAD3. In animal models, knockdown of HNRNPC effectively inhibits keloid growth [151]. At the protein level, post-translational modifications dictate effector protein stability and spatial dynamics. As a primary environmental trigger, hypoxia promotes the global level of protein SUMOylation. SUMOylation directly covalently modifies the HIF-1α protein and enhances its stability, while simultaneously enhancing the activity of the TGF-β/SMAD signaling pathway [152]. The SUMO1-modulated nucleocytoplasmic transport regulatory protein RanGAP1 facilitates the dissociation of the nuclear export receptor CRM1 from Smad4, resulting in the aberrant accumulation of Smad4 within the nucleus [153]. Overall, epigenetic regulation is the central apparatus that encodes the persistent fibrotic state in keloids. This system ensures the sustained and amplified output of TGF-β signaling and facilitates the intercellular spread of pathological information.

## 7. Therapeutic Insights

Keloids represent a highly challenging fibroproliferative skin disorder. These lesions generate cosmetic impact and substantial physical discomfort, including chronic pruritus and pain, which leads to a severe psychological burden for patients. For the treatment of keloids, early prevention should involve the use of silicone preparations. For existing lesions, the preferred treatment is the injection of glucocorticoids combined with 5-fluorouracil. For larger or stubborn keloids, surgical removal followed by immediate radiotherapy is necessary to effectively control recurrence. All these treatment plans emphasize individualized formulation and long-term management [154]. However, Traditional interventions frequently fall short in achieving long-term remission. They are severely limited by unacceptably high recurrence rates, often exceeding 50% when surgical excision or corticosteroid injections are administered as monotherapies [155,156]. These limitations suggest an unmet clinical need to develop more effective and targeted treatments.

### 7.1. Navigating the TGF-β Network with Multi-Targeted Strategies

While direct inhibition of TGF-β or its receptors has shown promise in various fibrotic conditions, the risk of systemic side effects due to TGF-β’s pleiotropic physiological roles remains a major concern [157]. The complexity and redundancy of TGF-β signaling in keloids necessitate a therapeutic shift from solely targeting the canonical Smad pathway to multi-targeted strategies (Table 2). 

Drug Repurposing. Repurposing approved medications offers rapid clinical translation due to their established safety profiles. Success depends on identifying compounds targeting multiple fibrotic pathways simultaneously. Clinically used kinase inhibitors, sorafenib, demonstrate this polypharmacology, which simultaneously antagonizes the TGF-β/Smad and MAPK/ERK axes [158]. The approved antifibrotic agent, nintedanib, concurrently inhibits the phosphorylation of Smad3 and key non-Smad effectors, including ERK, p38, JNK, and STAT3 [159]. And lapatinib can also simultaneously target the upstream receptors ErbB1/ErbB2 and the downstream TGF-β1/Smad2/3/Erk/Akt signaling axis [160]. Beyond oncology, other drug classes show similar broad-spectrum activity. The antimalarial drug artesunate has been confirmed to effectively inhibit the proliferation of KFs, a mechanism closely related to targeting the IRE1α/XBP1, PI3K/AKT/mTOR, and TGF-β/Smad signaling pathways [64,161]. The broad-spectrum antiviral agent Remdesivir’s efficacy in alleviating skin fibrosis through dual inhibition of TGF-β1/Smad and PI3K/Akt/mTOR pathways [63]. And melatonin simultaneously inhibits both the cAMP/PKA/Erk and Smad signaling pathways via its membrane receptor MT2 [162]. While drug repurposing offers rapid clinical translation, the complexity of keloids demands the pleiotropic potential of natural products.

Natural Products. Complementing drug repurposing, natural products offer a rich source of multi-target agents with favorable toxicity profiles, suitable for long-term modulation of complex diseases. Ginsenoside Rg3 and the flavonoid Glabridin both concurrently inhibit the TGF-β/Smad pathway alongside major non-Smad routes (ERK and PI3K/Akt, respectively) [163,164]. Asiaticoside presents a particularly comprehensive mode of action. It inhibits the classical pro-fibrotic axis (downregulating TGF-β1, upregulating Smad7), suppresses inflammatory non-Smad signaling (downregulating IL-1β, IL-6), and simultaneously activates the PPAR-γ [165]. Asiaticoside also blocks the GDF-9/MAPK/Smad pathway to control KFs’ proliferation [166]. Other natural compounds like daidzein inhibits TGF-β1/Smad signaling by targeting the pyruvate kinase M2 isotype (PKM2) [167]. And alpinetin broadly restrains fibrosis induced by TGF-β1 in human primary dermal fibroblasts [80]. 

Targeting Hub Molecules. Finally, targeting hub molecules is another important strategy for achieving multi-pathway coordinated intervention. Tropisetron exemplifies this elegant approach. By activating α7nAChR, it exerts a dual-inhibitory effect on TGF-β/Smad and NF-κB/TNF-α pathways, suppressing both fibrogenesis and inflammation. This regulatory cascade restores ECM homeostasis by modulating the collagen/MMP balance, ultimately reducing the scar area by approximately 40% [21]. Therefore, the multi-target coordinated intervention targeting multiple key nodes in the TGF-β signaling network represents a highly prospective treatment strategy for keloids.

### 7.2. Advanced and Targeted Therapeutic Modalities 

Microenvironment and Cellular Reprogramming. The ideal anti-fibrosis treatment is to reprogram pathological healing into normal repair, and reprogramming around the TGF-β network presents different patterns. Researchers found that injecting extracellular vesicles (MSC-EVs/UCB-EVs) and pH-responsive hydrogel (PVA-BA-SAB) in situ can simultaneously reduce TGF-β signal and increase Wnt signal. This restores the dynamic balance of key signals, which helps to heal and reduce scars [168,169]. A more direct approach involves orchestrating a conversion of cellular identity. Botulinum Toxin A, delivered via intralesional injection, activates the antagonistic BMP4/Smad1/5/8 pathway, inducing keloid myofibroblast differentiation towards adipocytic characteristics [170]. Targeting CTGF via locked nucleic acid-modified antisense oligonucleotides effectively suppresses TGF-β1 signaling. This silencing strategy yields robust anti-scarring effects [171]. Likewise, neutralizing monoclonal antibodies targeting the matricellular protein FSTL1 can reduce cellular reactivity to TGF-β1, offering a new biological agent strategy [172]. Furthermore, metabolic rewiring serves as the indispensable substrate that sustains the aberrant TGF-β signaling. Metabolic reprogramming through methionine restriction attenuated scar formation in HS fibroblasts by downregulating TGF-β-SMAD, STAT, and AKT/mTOR pathways [173]. However, realizing the full potential of microenvironmental reprogramming is fundamentally hindered by the dense physical barricade of the keloid stroma.

Advanced Delivery Systems. To overcome the skin barrier and achieve cell targeting, advanced delivery systems are required. PLGA nanoparticles loaded with the PPAR-γ agonist pioglitazone and stem cell-derived exosomes delivering miR-194 enable sustained drug release at the lesion site following direct intralesional injection. This combined strategy has shown superior anti-scar efficacy in animal models [174,175]. Besides, spatiotemporal co-delivery of IL-10 and decorin genes via biomaterial scaffolds (implanted directly into the wound bed) synergistically suppresses TGF-β1/β2 expression in the rabbit ear HS model [176]. The frontier of delivery is exemplified by platforms like orthogonal upconversion supramolecular microneedle arrays (OUSMNs). Precise anti-fibrotic therapy was achieved via transdermal drug delivery with photo-controlled targeted activation of singlet oxygen. This approach synergistically inhibited the PI3K/AKT/mTOR pathway while inducing ferroptosis [177]. Advanced delivery systems breach the dense fibrotic barrier, ensuring localized therapeutic accumulation with minimal off-target toxicity.

Physical and Energy-Based Modalities. Beyond mere delivery, physical and energy-based modalities provide a non-pharmacological dimension to network disruption. Carbon ion radiotherapy exerts dual anti-fibrotic effects in animal models. Beyond directly inhibiting the TGF-β/SMAD pathway, it also induces immunogenic cell death and drives macrophages towards the anti-fibrotic M1 phenotype [178]. Photodynamic therapy (PDT), using agents like Hypocrellin A or Phenalen-1-one, cooperatively inhibits the TGF-β/Smad and ERK pathways, disrupts cytoprotective autophagy, and reduces angiogenesis [179,180]. Moreover, regulation of the physical microenvironment, such as elongation of KFs mediated by aligned microgrooves, can prevent TGF-β1 expression and Smad/ERK signal activation, effectively reversing the fibrotic phenotype [181]. 

Cellular Therapies. Finally, cellular therapies transcend static molecular inhibition. Particularly, ADSCs secrete factors like TSG-6 (via the ROS/NF-κB axis) and IGFBP-7 to suppress the TGF-β/Smad and BRAF/MEK/ERK pathways, respectively. Local delivery of these cells or their secreted factors has been achieved through perilesional or intralesional injection in preclinical models [53,182].

### 7.3. Translating Mechanisms to the Clinic

Recent clinical trials are increasingly shifting away from broad, non-specific immune suppression toward precision medicine and regenerative approaches. These include modulating the TGF-β signaling axis through small interfering RNAs (NCT02079168) and microRNA mimics [183]. While early clinical trials of recombinant TGF-β3 (NCT00836147) faced delivery hurdles, recent advances in mRNA-engineered cell therapies have revitalized this axis by enabling precise cytokine expression to reprogram the fibrotic microenvironment [120]. Furthermore, clinical evaluations are actively exploring the efficacy of monoclonal antibodies targeting Th2-driven inflammation [184], anti-angiogenic agents, synthetic peptide analogs, and advanced cell-based therapies, such as MSCs (NCT05939817, NCT04553159) [185,186]. A comprehensive summary of these promising investigational drugs and biological modalities is provided in Table 3. 

In brief, targeting the complex TGF-β non-Smad network requires a practical, two-step strategy. Combination therapy is the most accessible choice, using advanced local delivery systems to block multiple non-Smad cascades. This provides fast symptom relief. Nevertheless, symptom control is not a cure. To truly prevent recurrence, we must look to precision nucleic acids and advanced cell therapies. These tools can directly silence core non-Smad hubs. They do not just mask symptoms but fundamentally reprogram the fibrotic microenvironment.

## 8. Conclusions and Future Perspectives

**Figure 4:**
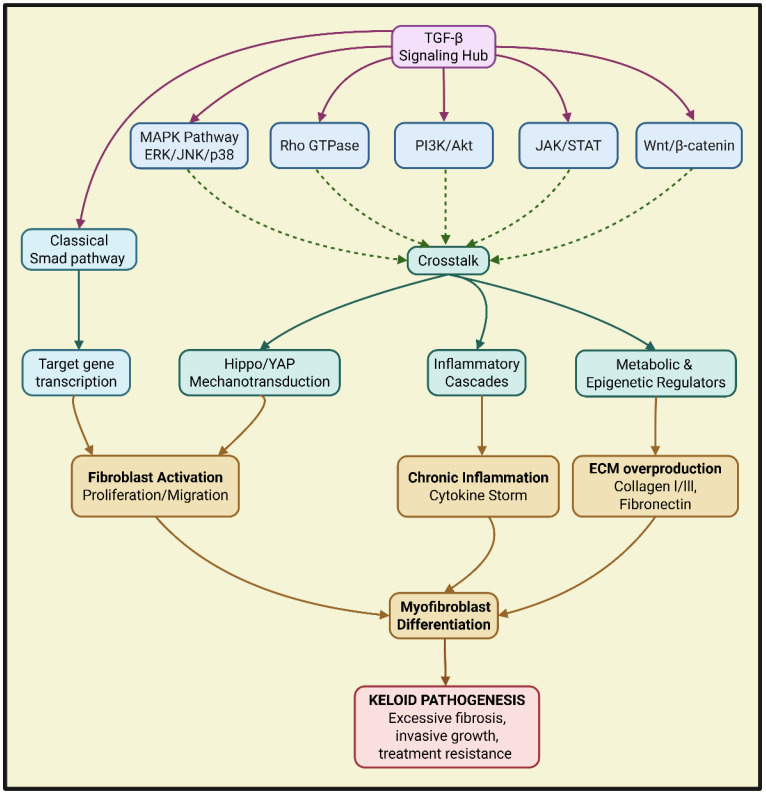
The integrative role of the TGF-β signaling network in the pathogenesis of keloids. As the core hub of fibrosis, TGF-β drives the molecular network of keloid occurrence and development through the synergy and crosstalk of the classical Smad pathway and multiple non-Smad pathways. Created with BioRender.com. Abbreviations: ERK, extracellular signal-regulated kinase; Hippo/YAP, Hippo/Yes-associated protein; JAK/STAT, Janus kinase/signal transducer and activator of transcription; JNK, c-Jun N-terminal kinase; p38, p38 mitogen-activated protein kinase; PI3K/AKT, phosphoinositide 3-kinase/protein kinase B; Rho GTPase, Rho guanosine triphosphatase; Wnt, Wingless/Integrated. Created with BioRender.com. https://BioRender.com/gn8a6ni (accessed on 29 March 2026).

Keloids with aggressive fibroblast overgrowth and excessive ECM accumulation represent a substantial clinical obstacle in dermatological and plastic surgery [187]. TGF-β is widely regarded as the pivotal orchestrator of keloid pathogenesis. The traditional focus on its canonical Smad-dependent pathway has proven insufficient for developing universally effective and safe therapeutic interventions. This review has systematically deconstructed the prevailing research, arguing that the involvement of TGF-β in keloid formation processes cannot be confined to the classical Smad pathway. Instead, we posit that TGF-β acts as a central processing unit within a dynamic and self-reinforcing pathological network, as shown in Figure 4. This network engages in extensive crosstalk with key developmental (MAPK, PI3K/Akt), mechanical (Hippo/YAP), and inflammatory (JAK/STAT, NF-κB) pathways and exhibits intrinsic functional antagonism between its own pro-fibrotic (TGF-β1/β2) and anti-fibrotic (TGF-β3) isoforms. These pathways collectively drive fibroblast growth, migration, apoptosis resistance, and excessive ECM deposition. Factors like mechanical tension, hypoxia, immune cell infiltration, and ncRNAs further modulate these interactions, contributing to the persistent and aggressive nature of keloids. A comprehensive appreciation of the TGF-β signaling network intricacy suggests that future therapeutic interventions require a systematic approach, including multi-targeting, reprogramming, and advanced administration methods. The timing of the intervention is also crucial. Xu et al. elucidated that the autophagic activity of fibroblasts positively regulates TGF-β1/Smad signaling by degrading SQSTM1/p62, acting as an upstream permissive switch for this pathway. This emphasizes that intervention in the TGF-β network must consider its dependence on the underlying cellular state [188]. Together, these findings suggest that TGF-β functions as a context-dependent signaling hub and has divergent roles for its isoforms. So future therapeutic strategies must be dynamically tailored to these specific pathological contexts.

The core challenge in treating keloids lies in their extremely high recurrence rate. Even with combined radiotherapy, it is difficult to completely control them. Injections and lasers are invasive and costly, while emerging therapies such as stem cells are immature. There are unresolved issues, including epigenetic memory in the mechanism and the lack of targeted drugs. Therefore, many blank areas remain to be investigated. For example, exploration of the precise crosstalk between non-Smad pathways and TGF-β signaling in KFs will require rigorous experimental designs through targeted pharmacological inhibition, genetic perturbations, and advanced biochemical assays. Similarly, explicating the distinct functions of individual TGF-β isoforms necessitates the application of specific tools, such as neutralizing antibodies, selective recombinant proteins, and conditional knockout murine models. Single-cell omics have also revealed novel fibroblast subpopulations with unique non-Smad signaling profiles. The functional significance of these distinct cells must be fully established to identify actionable therapeutic targets. This translational step can potentially be achieved through high-throughput drug screening and patient-derived organoid models.

Translating TGF-β network findings into clinical practice requires moving beyond single-target inhibition. Dual targeting of Smad and non-Smad pathways is a feasible combined strategy. However, a balance must be struck between efficacy and safety, as these pathways are also involved in maintaining skin homeostasis. Another approach is to reprogram the cell state to indirectly inhibit the TGF-β signal. Systemic administration has the risk of off-target effects, while local administration, including injectable hydrogels, microneedle patches, or controlled-release scaffolds enable sustained, site-specific treatment. Biomarkers based on TGF-β subtype profiles, non-Smad activation status, or fibroblast subpopulations can help identify patients suitable for multi-targeted treatment. In conclusion, from linear inhibition to systemic reprogramming is the essential next step inspired by enhanced insight into TGF-β’s true complexity.

## Figures and Tables

**Table 2:** Multitargeted therapeutic agents for keloids focusing on TGF-β/non-Smad pathways.

Strategies	Agents	Targets	Model	Outcomes	Refs
Drug Repurposing	Sorafenib	TGF-β/Smad and MAPK/ERK	HKFs; HKEs	Reduced cell migration, angiogenesis, and collagen accumulation.	[158]
	Nintedanib	Smad3 phosphorylation, ERK, p38, JNK, STAT3	KFs; KEs	Downregulated COL1, COL3, FN, α-SMA, and CTGF; Inhibited collagen deposition and microvessel formation.	[159]
	Lapatinib	ErbB1/ErbB2, TGF-β1/Smad2/3/Erk/Akt	KFs; KXM; BLM model	Reduced expression of α-SMA, COL1, and FN; Attenuated skin thickness, collagen deposition, and hydroxyproline content.	[160]
	Artesunate	IRE1α/XBP1, TGF-β1	HKFs	Induced G1 phase cell cycle arrest.	[161]
	Remdesivir	TGF-β1/Smad and PI3K/Akt/mTOR	KFs; KXM	Decreased α-SMA, COL1, and FN expression; Alleviated skin fibrosis and reduced xenograft weight.	[63]
	Melatonin	cAMP/PKA/Erk and Smad via MT2	KFs	Promoted apoptosis; Synergistic inhibition with 5-FU on Akt, mTOR, Erk, and Smad pathways.	[162]
Natural Products	Ginsenoside Rg3	TGF-β/Smad and ERK	KFs; KEs	Inhibited fibroblast proliferation, migration, and invasion; reduced collagen deposition and angiogenesis.	[163]
	Glabridin	PI3K/Akt and TGF-β1/SMAD	HKFs; REHS model	Inhibited scar hyperplasia, inflammation, and collagen deposition.	[164]
	Asiaticoside	TGF-β1/Smad7, IL-1β/IL-6, PPAR-γ; GDF-9/MAPK/Smad	KFs; REHS model	Dose-dependent inhibition of scar hyperplasia.	[165,166]
	Daidzein	PKM2, TGF-β1/Smad	KFs; BLM model; KXM	Alleviated skin fibrosis and transplant tumor growth in vivo.	[167]
	Alpinetin	TGF-β1, β-catenin	HDFs	Inhibited TGF-β1-induced cell proliferation and migration.	[80]
Targeting Hub Molecules	Tropisetron	α7nAChR, TGF-β/Smad, NF-κB/TNF-α	KFs; RISM	Reduced scar area, improved collagen arrangement.	[21]

Abbreviations: α7nAChR, alpha-7 nicotinic acetylcholine receptor; BLM, bleomycin; CTGF, connective tissue growth factor; GDF-9, growth differentiation factor 9; HDFs, human dermal fibroblasts; HKEs, human keloid explants; HKFs, human keloid fibroblasts; IRE1α, inositol-requiring enzyme 1 alpha; KEs, keloid explants; KFs, keloid fibroblasts; KXM, keloid xenograft model; PKM2, pyruvate kinase M2; PPAR-γ, peroxisome proliferator-activated receptor gamma; REHS, rabbit ear hypertrophic scar; RISM, rat incisional scar model; XBP1, X-box binding protein 1.

**Table 3:** Overview of Promising Investigational Drugs and Targeted Therapies for Keloids in Clinical Trials.

Intervention (Drug)	Mechanism of Action/Molecular Target	Study Phase	ClinicalTrials.gov Identifier
Ritlecitinib	JAK3/TEC kinase inhibitor (Blocks inflammatory cytokine signaling)	Phase 2	NCT06373458
Dupilumab	IL-4Rα antagonist (Inhibits Th2-mediated fibrotic inflammation)	Phase 2/4	NCT05128383, NCT04988022
STP705	Dual TGF-β1 and COX-2 siRNA (Suppresses fibrogenesis and inflammation)	Phase 2	NCT04844840
Remlarsen (MRG-201)	microRNA-29 mimic (Inhibits ECM expression and fibroplasia)	Phase 2	NCT03601052
Bevacizumab	Anti-VEGF monoclonal antibody (Inhibits pathological angiogenesis)	Phase 4	NCT07014280
Pirfenidone (8% gel)	Broad anti-fibrotic agent (Repurposed; modulates TGF-β production)	Phase 3	NCT06909812
RXI-109	CTGF targeted RNAi	Phase 2	NCT02079168
Avotermin	Recombinant human TGF-β3 (Promotes regenerative vs. fibrotic healing)	Phase 1/2	NCT00469235, NCT00836147
MSC Therapy (UC-MSCs/ADSCs)	Immunomodulation and anti-fibrotic paracrine signaling	Phase 2/4	NCT05939817, NCT04553159

Table data sourced from ClinicalTrials.gov. Abbreviations: JAK, Janus kinase; IL, interleukin; TGF-β, transforming growth factor-beta; COX-2, cyclooxygenase-2; VEGF, vascular endothelial growth factor; CTGF, connective tissue growth factor; siRNA, small interfering RNA; RNAi, RNA interference; HSP20, heat shock protein 20; MSC, mesenchymal stem cell; UC, umbilical cord; ADSC, adipose-derived stem cell.

## Data Availability

No new data were created or analyzed in this study. Data sharing is not applicable to this article.

## Abbreviations

The following abbreviations are used in this manuscript:

TGF-β	transforming growth factor-beta
MAPK	mitogen-activated protein kinase
PI3K	phosphatidylinositol 3-kinase
Akt	protein kinase B
Rho GTPase	Rho family of GTPases
Wnt	wingless/integrated
STAT	signal transducer and activator of transcription
ECM	extracellular matrix
NEDD4L	neural precursor cell expressed developmentally down-regulated protein 4-like
YY1	Yin Yang 1
HK2	hexokinase 2
Th	T helper cell
CCL2	C-C motif chemokine ligand 2
S100A7	S100 calcium-binding protein A7
LTBP	latent transforming growth factor beta binding protein
KFs	keloid fibroblasts
MMT	mesothelial-to-mesenchymal transition
circRNA	circular RNA
CTRP3	C1q/tumor necrosis factor-related protein 3
EMT	epithelial-mesenchymal transition
ERK	extracellular signal-regulated kinase
p38	p38 mitogen-activated protein kinase
TβR	transforming growth factor-beta receptor
SSXS	synovial sarcoma X breakpoint
PAI-1	plasminogen activator inhibitor-1
CXCL12	C-X-C motif chemokine ligand 12
CXCR4	C-X-C chemokine receptor type 4
MMPs	matrix metalloproteinases
HIF-1α	hypoxia-inducible factor 1-alpha
IL-10RA	interleukin-10 receptor alpha
miR	microRNA
CRIF1	cytokine receptor-like factor 1
SMURF2	SMAD specific E3 ubiquitin protein ligase 2
CEMIP	cell migration-inducing protein
SPARC	secreted protein acidic and rich in cysteine
CD	cluster of differentiation
FOXM1	forkhead box M1
TWIST1	twist-related protein 1
FOXO4	forkhead box O4
TRAF3IP2	TRAF3 interacting protein 2
MEF2A	myocyte enhancer factor 2A
USP11	ubiquitin specific peptidase 11
TSG-6	tumor necrosis factor-stimulated gene-6
SNAI2	snail family transcriptional repressor 2
Shc	Src homology 2 domain containing-transforming protein 1
Grb2	growth factor receptor-bound protein 2
SOS	Son of Sevenless
Ras	Rat sarcoma (virus)
EGFR	epidermal growth factor receptor
ADAM17	ADAM metallopeptidase domain 17
HOXC6	homeobox C6
JNK	c-Jun N-terminal kinase
TAK1	transforming growth factor-beta-activated kinase 1
TIMPs	tissue inhibitors of metalloproteinases
OMD	osteomodulin
BMP	bone morphogenetic protein
Runx2	runt-related transcription factor 2
mTOR	mammalian target of rapamycin
HS	hypertrophic scar
UCHL1	ubiquitin C-terminal hydrolase L1
IGF-1	insulin-like growth factor-1
PGK1	phosphoglycerate kinase 1
SCUBE3	signal peptide-CUB-EGF domain-containing protein 3
NF-κB	nuclear factor kappa B
APJ	apelin receptor
CREB1	cAMP responsive element binding protein 1
RhoA	Ras homolog family member A
Rac1	Ras-related C3 botulinum toxin substrate 1
Cdc42	cell division cycle 42
ROCK	Rho-associated coiled-coil containing protein kinase
MRTF-A	myocardin-related transcription factor A
α-SMA	alpha-smooth muscle actin
EN1	engrailed homeobox 1
YAP	Yes-associated protein
TAZ	transcriptional coactivator with PDZ-binding motif
HPDFs	human primary dermal fibroblasts
TCF	T-cell factor
LEF	lymphoid enhancer-binding factor
GSK-3β	glycogen synthase kinase-3 beta
TMEM88	transmembrane protein 88
FGF	fibroblast growth factor
Notch	neurogenic locus notch homolog protein
JAK	Janus kinase
SRC	proto-oncogene tyrosine-protein kinase Src
c-ABL	ABL proto-oncogene 1, non-receptor tyrosine kinase
BRD4	bromodomain-containing protein 4
KLF	Kruppel-like factor
TSP1	thrombospondin 1
MyD88	myeloid differentiation primary response 88
ILK	integrin-linked kinase
PPARs	peroxisome proliferator-activated receptors
Axl	AXL receptor tyrosine kinase
OPA1	optic atrophy 1
TGM2	transglutaminase 2
RREB1	ras responsive element binding protein 1
PDGFB	platelet-derived growth factor subunit B
SNAI1	snail family transcriptional repressor 1
INO80	INO80 complex ATPase subunit
ITGAV	integrin subunit alpha V
GAB1	GRB2-associated binding protein 1
TGFBR2	transforming growth factor beta receptor 2
NOX4	NADPH oxidase 4
ROS	reactive oxygen species
FBLN4	fibulin 4
CPEB	cytoplasmic polyadenylation element binding protein
YBX1	Y-box binding protein 1
SPRY1	sprouty RTK signaling antagonist 1
ceRNA	competing endogenous RNA
IGFBP2+	insulin-like growth factor binding protein 2 positive
POSTN+	periostin positive
m6A	N6-methyladenosine
SUMO	small ubiquitin-like modifier
PKM2	pyruvate kinase M2
cAMP	cyclic adenosine monophosphate
FSTL1	follistatin-like 1
PLGA	poly(lactic-co-glycolic acid)
